# Laser Surface Microstructuring of Biocompatible Materials Using a Microlens Array and the Talbot Effect: Evaluation of the Cell Adhesion

**DOI:** 10.3390/ma10020214

**Published:** 2017-02-22

**Authors:** María Aymerich, Daniel Nieto, Ezequiel Álvarez, María T. Flores-Arias

**Affiliations:** 1 Photonics4Life Research Group, Departamento de Física Aplicada, Facultad de Física, Universidade de Santiago de Compostela, Santiago de Compostela 15782, Spain; maria.aymerich@usc.es (M.A.); daniel.nieto@usc.es (D.N.); 2 Instituto de Investigación Sanitaria de Santiago de Compostela (IDIS), Complexo Hospitalario Universitario de Santiago de Compostela (CHUS) SERGAS, Santiago de Compostela 15706, Spain and CIBERCV, Madrid, Spain; ezequiel.alvarez.castro@gmail.com

**Keywords:** laser microstructuring, Talbot effect, biocompatible materials, cell adhesion

## Abstract

A laser based technique for microstructuring titanium and tantalum substrates using the Talbot effect and an array of microlenses is presented. By using this hybrid technique; we are able to generate different patterns and geometries on the top surfaces of the biomaterials. The Talbot effect allows us to rapidly make microstructuring, solving the common problems of using microlenses for multipatterning; where the material expelled during the ablation of biomaterials damages the microlens. The Talbot effect permits us to increase the working distance and reduce the period of the patterns. We also demonstrate that the geometries and patterns act as anchor points for cells; affecting the cell adhesion to the metallic substrates and guiding how they spread over the material.

## 1. Introduction

The surface modification of different materials is a widely-studied procedure due to the numerous applications that it presents in industry [1] and in the biomedical field [2]. The microstructuring of substrates revieves high interest in the industry because of the potential to alter tribological properties of materials, such as wettability or friction [3,4]. It also has several biomedical applications, like tissue engineering [5], prosthesis [6], or biosensing [7]. It has been reported by several authors that physical aspects like roughness of the substrate [8] or its elasticity [9] affects the cell adhesion, depending on the type of the cell and the material employed for the culture [10]. Another determinant physical factor in cell culture substrates is its topography [11,12], which can be modified at the micron or nano scale [13,14] by different techniques. Titanium and tantalum are two of the most widely biocompatible metals employed as cell substrates due to their applications in orthopaedics and cardiovascular implants [15,16]. Cell adhesion is a very important factor for the success of these prostheses and there are numerous works that demonstrate that the modification of the surface of tantalum and titanium at the micron scale leads to a higher cell attachment [17,18,19,20].

Different techniques for surface microstructuring have been reported over recent years. Some examples are photolithography [21] or chemical etching [22], but among all of them, laser ablation is the most promising. Due to its versatility, speed, and non-contact nature, this technique presents great advantages over the others [23]. In particular, femtosecond lasers have been used for surface microstructuring of titanium plates, improving the compatibility of the material [24]. Some of the authors have demonstrated the use of the foci of a microlens array to multistructure surfaces by laser direct writing of stainless steel, copper, polymer, and aluminium [25]. In this situation, the ablative material is placed at the focal length from the array, which is illuminated by the laser beam. The energy is concentrated in each focus of the array that leads to a very effective process of microstructuring. Therefore, microlens arrays are well-suited for improving the efficiency of laser texturing techniques as well as the cost involved in the process [26].

However, it has been shown in previous works [25] that this procedure leads to a rapid deterioration of the microlenses due to the expelled materials during ablation that reach the array because of the short value of the focal length, causing an irreversible damage and a reduced useful life of the microlens array. Some of the authors have reported the Talbot effect as a solution to overcome this problem, increasing the distance between the array and the target [27]. This phenomenon consists of the repetition of the complex amplitude distribution of a periodic object along the light propagation axis when it is illuminated by a coherent beam [28]. The foci of the microlenses can be considered as a periodic object that repeats its image at several integer distances from the array. These Talbot positions depend on the wavelength of the beam, the kind of illumination, and the period of the object. The image can appear at a fractional value of the Talbot distance and with a minor period from the original, proportional to the fraction, which is called fractional Talbot effect [29]. The capability to microstructure different substrates using the Talbot effect and laser irradiation has been studied some years ago [30,31], and we aim to microstructure biocompatible material surfaces—such as titanium and tantalum—by a direct laser writing technique using the foci of an array of microlenses and the integer and fractional Talbot effect to avoid the deterioration of the array and to obtain patterns with a minor period.

In this work, we study the cell adhesion over biocompatible materials—in particular titanium and tantalum—when their surfaces are microstructured using the Talbot effect and a nanosecond laser is presented. The advantage of using lasers in the nanosecond regime instead of the femtosecond regime is that the first one is commonly implemented in industry. Section 2 is devoted to the materials and methods employed in this work. Section 3 presents the results of surface microstructuring and the behavior of endothelial cells when they are seeded over the structured materials. Section 4 shows the discussion of the results, and Section 5 presents the main conclusions of the work.

## 2. Materials and Methods

In order to microstructure the biocompatible materials, a Quanta-Ray Nd:YAG laser (Spectra Physics, Mountain View, CA, USA), in a Q-Switch regime with a pulse duration of nanoseconds operating on the second harmonic (λ = 532 nm) was employed. The laser parameters were a pulse width of 20 ns at 50 Hz and *M*^2^ < 1.2. The targets employed were a foil of titanium (99.6% purity) of 0.7 mm thickness and another of tantalum (99.9% purity) of 0.5 mm thickness, provided by Goodfellow (Delson, QC, Canada). The samples were polished before the laser texturing in order to decrease the surface roughness with a Logitech PM2A polisher (Romanel-sur-Morges, Switzerland). Substrates had to be polished to ensure roughness did not influence cell attachment and to achieve a good quality microstructuring since their initial roughness values were comparable with the pattern’s depths. A Nikon MM-400 microscope (Tokyo, Japan) was used for the visual characterization of the samples.

The microlens array employed had 90 μm period and 1.04 ± 0.03 mm focal length, experimentally verified. These microlenses, made of fused silica, were fabricated by reactive-ion etching technique, with a diameter of 80 μm and a sag of 3 μm. A confocal microscope Sensofar PLμ 2300 (Barcelona, Spain) allowed us to obtain 3D images of them. The results presented were acquired using a 20x EPI microscope objective. To carry out the measurement of the roughness of the substrates, a Dektak^3^ Profilometer (Billerica, MA, USA) was employed. The average roughness (R_a_) was obtained using a scanline of 60 μm. The mean value of R_a_ was determined from 10 scans randomly distributed over a surface of 2 mm^2^. The detailed images of the fabricated structure were acquired using a scanning electron microscope FESEM Zeiss Ultra Plus (Oberkochen, Germny). A Zeiss EVO LS 15 scanning electron microscope (Oberkochen, Germany) were employed to obtain the SEM images of the cells over the microholes.

The identification of the Talbot images was done with the Nd:YAG laser mentioned above operating at continuous wave and using an acquisition image system composed by a 20x microscope objective and a CCD camera.

For the cell culture, human umbilical vein endothelial cells (HUVEC) were employed. These cells were obtained from umbilical cords donated after informed consent from the mothers. This protocol was approved by the Ethics Committee for Human Studies at Galicia (Spanish region) in accordance with the 1975 Declaration of Helsinki. HUVECs were isolated and cultured as previously described [32]. Cells were cultured in complete endothelial growth medium (EGM-2; Lonza, Basle, Switzerland) under standard cell culture conditions (37 °C temperature, more than 80% humidity and 5% CO_2_ level). They were stained with calcein AM (Invitrogen, Thermo Fischer Scientific, Waltham, MA, USA) for 4 min at 37 °C. This cell-permeant dye is converted to green-fluorescent calcein in live cells. After labelling, cells were washed twice in EGM-2 media to remove the excess calcein. The titanium and tantalum foils were sterilized in an autoclave (120 °C, 30 min) and immersed in EGM-2 media for 20 min before the cell deposition as a surface pre-treatment. Cells were seeded over titanium and tantalum substrates at a density 200,000 cells/1.5 mL and incubated for 17 h. After that, non-adherent cells were removed by gently washing the surfaces with EGM-2 and the patterns were observed under fluorescence microscopy, using a confocal microscope Leica TCS-SP8 (Wetzlar, Germany). Five random fields were counted per substrate area of interest and two different researchers made the count independently to get the experimental result. Experiments were conducted in triplicate and repeated at three different times. The mean value and the standard error mean were calculated with these data. A Student’s *t*-test was also performed to compare the data from the structured surfaces with those from the non-structured surfaces.

## 3. Results

### 3.1. Surface Multistructuring

In this subsection, a method for microstructuring the surfaces of biocompatible materials by a low-cost and effective hybrid method is presented. It is composed of a direct laser writing technique using a microlens array and the Talbot effect or self-image phenomenon. An introduction to the integer and fractional Talbot effect is shown. After the ablation of the substrates, an experimental validation of the Talbot distances is carried out.

#### 3.1.1. Integer and Fractional Talbot Effect

The Talbot effect is a diffraction effect that consists of the repetition of the image of a periodic object when it is illuminated by a coherent source of illumination. The repetition of the image along the longitudinal axis of propagation of light occurs without the need of optical elements, such as lenses or mirrors. It was demonstrated that not only periodic objects cause this phenomenon, the Talbot effect also occurs for objects that satisfy the Montgomery conditions [33]. Periodic objects are a subgroup of the Montgomery objects, but it must be indicated that periodic objects, such as the foci of a microlens array, are the most employed to observe this phenomenon [34]. These Talbot positions depend on several factors, such as the wavelength of the beam or the period of the object. Another factor is the kind of illumination that is employed, i.e., the shape of the wavefront that illuminates the periodic object. When a plane wave is chosen, the Talbot distances can be calculated by [28]

(1) $$z_{T}=n\frac{p^{2}}{\lambda }$$

where *z_T_* is the distance between the periodic object and the complex amplitude distribution, *n* is an real number, *p* is the period of the object, and λ is the wavelength of illumination. *n* can be an integer number (integer Talbot effect) or a fractional one (fractional Talbot effect). In this case, the image of the periodic object is repeated at a fraction value of the integer Talbot distance and the period *p_T_* is proportional to the period of the integer images [35].

(2) $$z_{T}=\frac{u}{v}\frac{p^{2}}{\lambda }$$

(3) $$p_{T}=\frac{p}{v}$$

where *u* and *v* are integer coprime numbers.

In this work, we consider the foci of a microlens array as a periodic object that repeats its complex amplitude distribution according to the integer and fractional Talbot effect. If a microlens array is employed to produce the Talbot effect, the value of the focal length of the array must be added to the Talbot distance in order to obtain the total distance between the object and the image [36]. The total distance is given by

(4) $$z=f_{ML}+z_{T}$$

where *f_ML_* is the focal length of the microlenses and *z_T_* is the Talbot distance, integer, or fractional.

#### 3.1.2. Identification of the Talbot Planes

**Figure 1:**
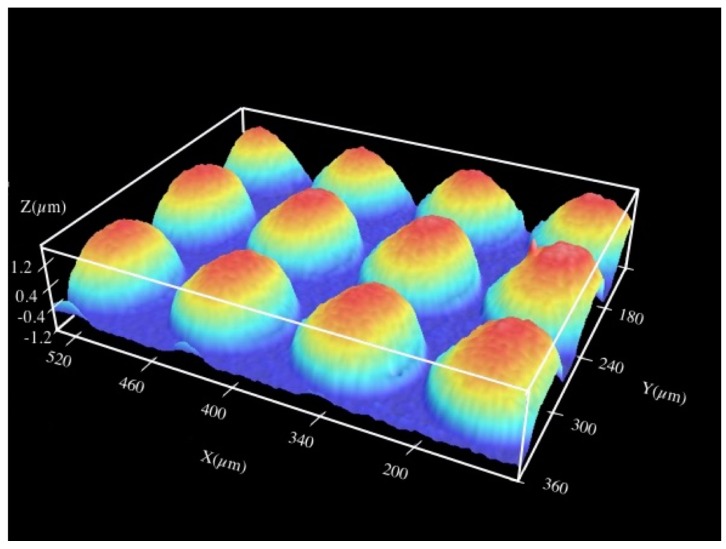
Confocal image of the microlens array with period of 90 μm.

Before carrying out the ablation of the targets, the Talbot planes of the foci that show a more uniform irradiance profile were chosen to work with them and it was verified that the images appear at the positions predicted by Equations (1) or (2). Figure 1 depicts a confocal image of the microlenses that are in this work.

**Figure 2:**
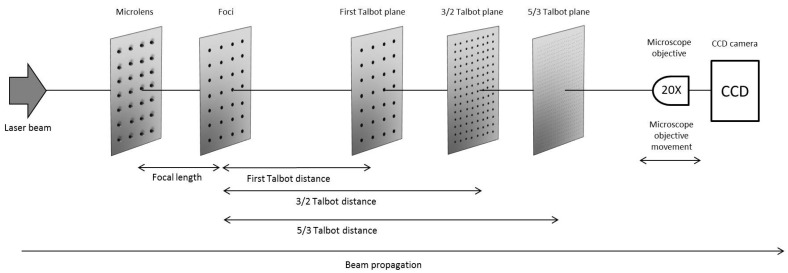
Experimental setup for the identification of the Talbot distances of the foci of a microlens array when it was illuminated by a Nd:YAG laser operating in a continuous wave. The image acquisition system for recording the Talbot images was conformed by a 20x microscope objective and a CCD camera.

The period of the microlenses was 90 μm and they had a focal length of *f_ML_* = 1.04 ± 0.03 mm, experimentally verified. The setup for the identification of the Talbot images of the foci of these microlenses is shown in Figure 2.

**Figure 3:**
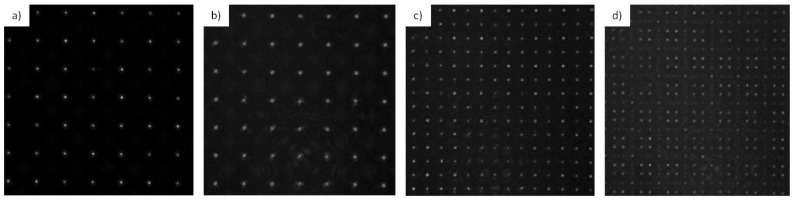
CCD-images recorded with the setup described in Figure 2 of the (**a**) foci of the microlenses; (**b**) first Talbot plane; (**c**) 3/2 Talbot plane; and (**d**) 5/3 Talbot plane. All images were taken at the same scale.

Figure 2 shows the experimental setup for the identification of the Talbot images of the foci of the microlens array. A Nd:YAG laser beam operating in a continuous wave at λ = 532 nm illuminated the microlens array. We assumed that the divergence of the beam was negligible, so the output of the laser was a plane wave and Equation (1) could be applied. The acquisition system consisted of a CCD camera and a microscope objective. The microscope objective was moved longitudinally along the propagation axis, forming image of the Talbot planes. The CCD camera records the intensity distribution. For this work, the first Talbot distance and the fractional Talbot distances 3/2 and 5/3 were chosen. These images occurred at theoretical positions of *z*_1_ = 16.27 ± 0.01 mm, *z*_3/2_ = 23.89 ± 0.01 mm, and *z*_5/3_ = 26.42 ± 0.01 mm, respectively, from the array. Figure 3 depicts the Talbot images recorded with the CCD camera.

In Figure 3 we can see the CCD-images of the Talbot images from the foci. These images were found at experimental positions of *z*_1_ = 16.21 ± 0.01 mm, *z*_3/2_ = 23.85 ± 0.01 mm, and *z*_5/3_ = 26.42 ± 0.01 mm, so it is shown that the theoretical positions are in good agreement with the experimental ones. We can see that the fractional plane that corresponds to the 5/3 Talbot image is less intense than the others. This is because the light was distributed between more points than in the other cases, resulting in less intense foci images than in the integer pattern. We will see that this fact will have repercussions on the ablation procedure. We can also observe that the period of the foci decreased in the fractional images, according to Equation (3).

#### 3.1.3. Titanium and Tantalum Ablation

**Figure 4:**
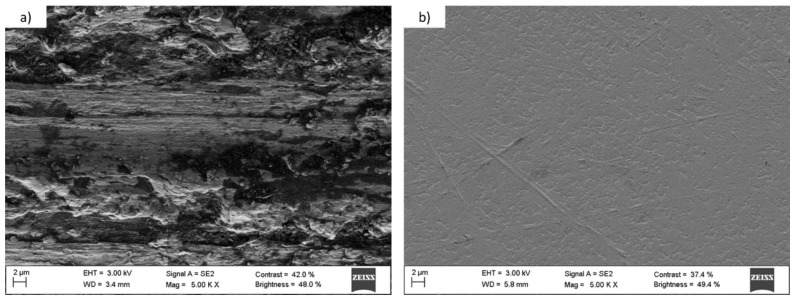
SEM images of the tantalum substrate (**a**) before and (**b**) after being polished. The roughness value decreased from 565.71 nm to 7.81 nm.

Once the Talbot planes were selected and verified experimentally, we proceed to the microstructuring of titanium and tantalum surfaces. Several studies demonstrate the influence of the substrate roughness on the cellular adhesion [37,38,39], so the roughness of both materials was reduced by polishing to similar values before the microstructuring to minimize the influence of the initial roughness of the surface. Figure 4 shows SEM images of the tantalum surface before and after the polishing.

**Figure 5:**
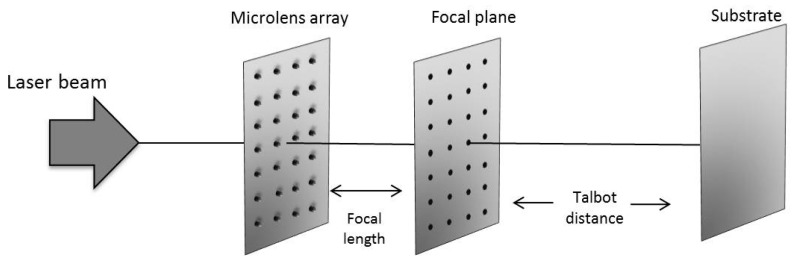
Experimental setup for performing the ablation of the substrates using the foci of a microlens array and a pulsed Nd:YAG laser. The material was placed at a Talbot distance from the foci.

Figure 4 shows the change of the tantalum surface after being polished. The non-polished tantalum surface had an average roughness (R_a_) of 565.71 ± 62.76 nm and it decreased to a value of 7.81 ± 0.91 nm. Titanium R_a_ was reduced from 639.42 ± 33.87 nm to 9.26 ± 1.39 nm. After polishing, both surfaces had a similar average roughness and a controlled microstructuring could be performed. The setup for the microstructuring is shown in Figure 5.

**Figure 6:**
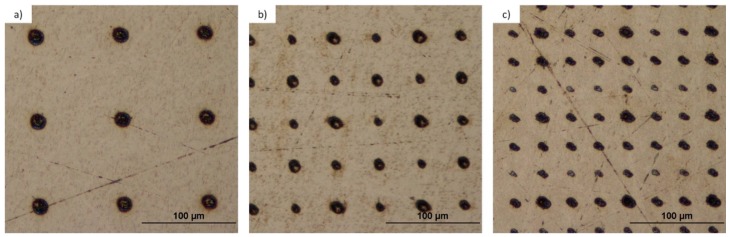
Optical microscope images obtained in reflected mode and bright field of the micropattern over a titanium foil of 0.7 mm thickness obtained after the ablation using the foci of the microlenses when the surface is placed at the (**a**) first Talbot distance; (**b**) 3/2 Talbot distance; and (**c**) 5/3 Talbot distance.

**Figure 7:**
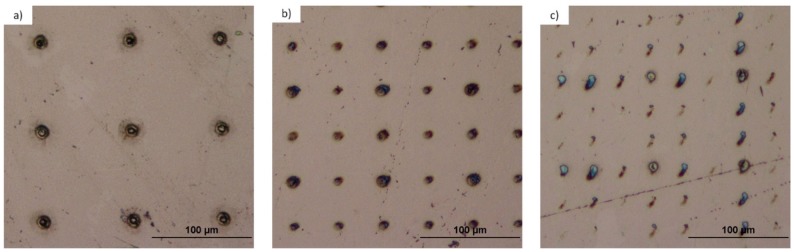
Optical microscope images obtained in reflected mode and bright field of the micropattern over a tantalum foil of 0.5 mm thickness obtained after the ablation using the foci of the microlenses when the surface is placed at the (**a**) first Talbot distance; (**b**) 3/2 Talbot distance; and (**c**) 5/3 Talbot distance.

Figure 5 depicts the experimental setup for the microstructing of targets using a microlens array and the Talbot effect. The laser employed was a Nd:YAG operating at Q-Switch mode with wavelength of λ = 532 nm, pulse duration of 20 ns, repetition rate of 50 Hz and energy per pulse of 450 μJ. The beam illuminates the microlens array and the substrate was placed at the Talbot distances, experimentally verified in the previous subsection. Figure 6 and Figure 7 show the microstructured titanium and tantalum surfaces, respectively. These microstructures were carried out at the first, 3/2, and 5/3 Talbot planes of the foci.

According to Equation (3), the original period of 90 μm remains the same for the first integer Talbot image and decreases to 45 μm at the 3/2 and to 30 μm at the 5/3 fractional Talbot images, respectively. As we can see in these figures, the period of the patterns was reduced as was expected. Table 1 summarizes the experimental values of different parameters of the structure of both substrates.

**Figure 8:**
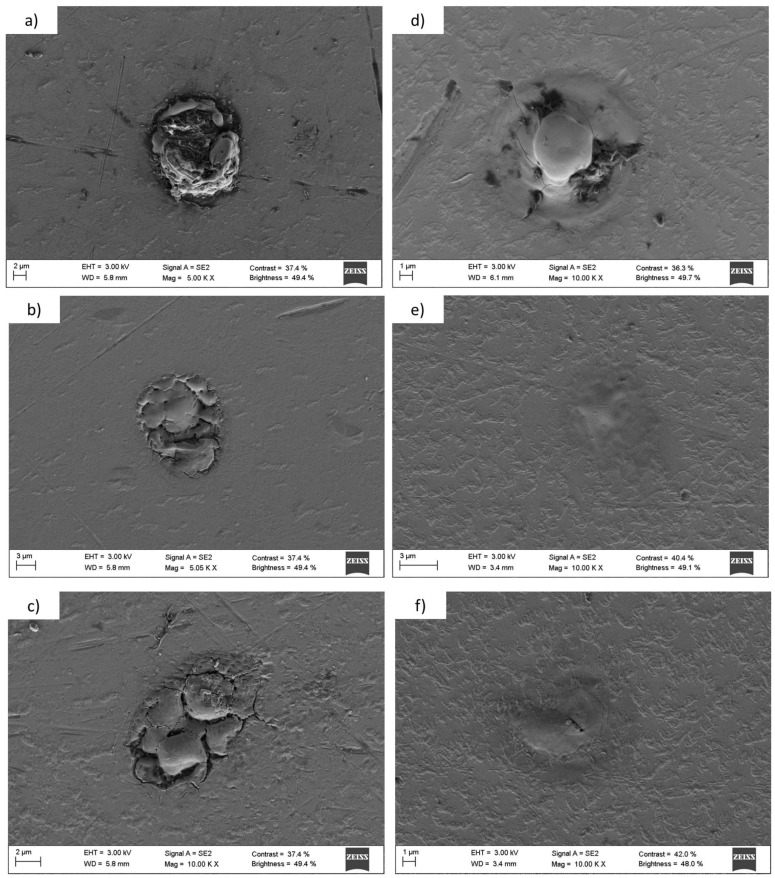
SEM images of the microholes fabricated with the microlenses and the Talbot effect. Figures (**a**–**c**) depict the patterns on titanium when the working distance was the first Talbot distance, 3/2, and 5/3, respectively; Figures (**d**–**f**) show the holes obtained on tantalum at the first, 3/2, and 5/3 self-image, respectively.

As expected, the integer planes led to deeper holes because each focus carried more energy than in the fractional case. The results depict that, working with the same laser parameters, less material was removed from the tantalum than from the titanium during the ablation process, as expected, because tantalum presents a higher hardness value than titanium. The exposure times were 8 s from the first Talbot plane, 40 s for the 3/2, and 360 s for the 5/3. The 5/3 needed more exposure time due to the fact that there was less energy per focus than in the case of the first Talbot image, since the light redistributes through the image. In fact, in the case of the tantalum, there was not enough energy per focus to ablate the substrate homogeneously. We tested if the exposure time for tantalum was increased, no more material would be removed and no better patterns would be obtained. There is a maximum quantity of material that can be removed by ablation using these laser parameters and the Talbot effect. Figure 8 shows SEM images of a detail of every micropattern.

In Figure 8, we can observe a detail of a randomly chosen microhole of each of the fabricated microstructures. As it was predicted, the microhole size decreases as the period of the Talbot plane does so. We can appreciate the difference in the ablation between tantalum and titanium. More material was removed during the ablation process of titanium than in the tantalum process, leading to less deep structures in this last case. The spot diameters of the different planes over titanium and tantalum are indicated in Table 1.

### 3.2. Cell Behavior

**Figure 9:**
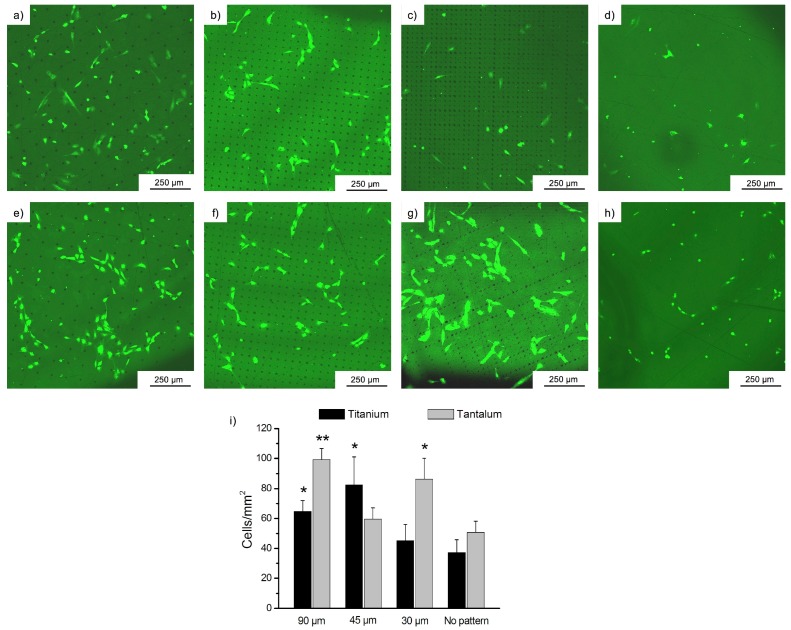
Images achieved by fluorescence microscopy of the endothelial cells over the different patterns after 17 h culture. Figures (**a**–**c**) show the cells attachment over titanium when the period of the structure is 90, 45, and 30 μm, respectively; In figures (**e**–**g**), we can see the cells spread on structured tantalum with periods 90, 45, and 30 μm, respectively; Figures (**d**,**h**) correspond to non-structured titanium and tantalum, respectively; Figure (**i**) shows a histogram that represents the number of cells per mm^2^ of material (mean ± standard error of the mean in vertical bars) over the different structured and non-structured surfaces. * *p* < 0.05 and ** *p* < 0.005 with respect to non-patterned surfaces.

Once the biocompatible materials were structured with the technique presented in the above subsection, we cultured Human Umbilical Vein Endothelial Cells (HUVECs) over them in order to observe the differences on the cell behavior and cell spreading when the material surface is flat and when it has a controlled pattern on the surface. Cells were seeded over the substrates and incubated for 17 h at standard conditions. After this time, the foils were washed to remove the non-adherent cells and the results were observed under fluorescence microscopy. Figure 9 shows these images.

**Figure 10:**
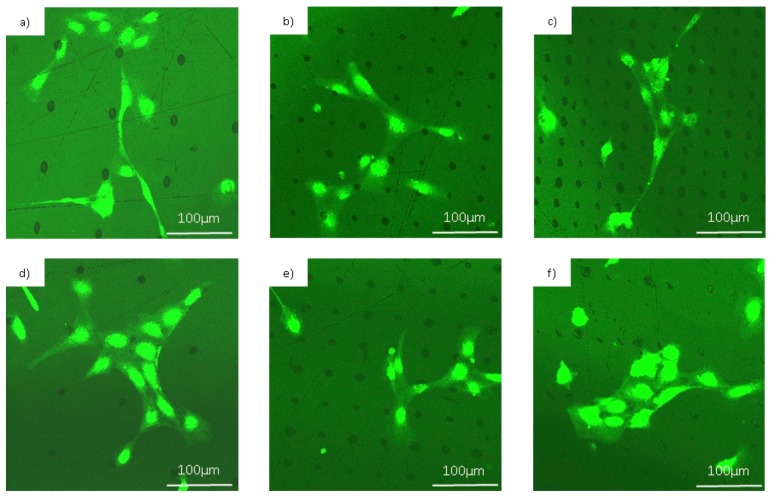
Fluorescence microscopy images of the endothelial cells over the different patterns after 17 h culture. (**a**–**c**) show the endothelial cell attachment to the holes fabricated on titanium with period 90, 45, and 30 μm, respectively; (**d**–**f**) show the cells on structured tantalum with pitch 90, 45, and 30 μm, respectively.

**Figure 11:**
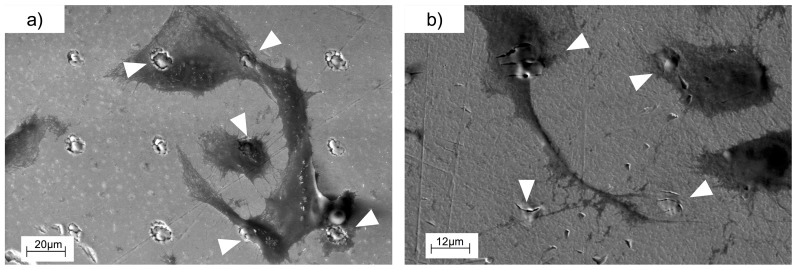
SEM images of the pattern of 45 μm on both (**a**) titanium and (**b**) tantalum where the cell guiding is shown. Arrowheads indicate the microholes fabricated that act as anchor points for cells.

In Figure 9, we can see how endothelial cells attached to the different patterned and non-patterned surfaces. As calcein is a viability indicator, we can say that HUVECs were alive in all cases but an interesting difference in the cell growth and spreading was observed between flat and structured surfaces. While in flat surfaces cells initially adhered randomly, in the patterned ones they attached to the holes rather than to the smooth substrate. These results will be discussed in Section 3. In Figure 10, detailed images of the adhesion of endothelial cells are shown in order to get a better appreciation of this phenomenon. Finally, we present SEM images of the cells spread over the patterned substrates in Figure 11.

In Figure 10 and Figure 11, we can clearly observe how the cells attached to the microholes fabricated with lasers and how they spread toward another anchor point. The attachment between the cells can also be appreciated.

## 4. Discussion

In this work, an effective and low-cost method to microstructure substrates by laser direct writing using the integer and fractional Talbot effect was presented. A microlens array was employed as the structure that repeated its image along the propagation axis and patterns of microholes with different periods and diameters were obtained. The ablated substrates were biocompatible materials (titanium and tantalum) and the cellular adhesion of endothelial cells to these microstructured surfaces was analyzed. In the first hours of cell culture over both materials, HUVECs mainly adhered to the laser-generated holes rather than to the non-structured surface. Cells also preferred to attach to another cell that was in contact with a microhole, and eventually spread to another structure, instead of adhering to the flat surface.

In Figure 9, we can see how the endothelial cells attached to the fabricated microholes that acted as anchor points for them. Figure 9i shows a graphic that represents the number of alive cells over the patterned and non-patterned surfaces. We can appreciate that there was certain cell adhesion to the flat surfaces, due to their biocompatible nature, but the number of cells attached was quantitatively less than in the microstructured surfaces. All the microstructured surfaces showed a tendency to improve cell adhesion. However, only on the patterns of 30 µm and 90 µm in tantalum, and on 45 µm and 90 µm in titanium, was cell adhesion statistically higher in comparison with non-structured surface. Whereas in non-structured surfaces the cell spread was totally random, we observed that cells attached to the structures fabricated with laser and then they spread and anchored around the holes. Comparing both titanium and tantalum, we could say that in general, tantalum seemed to present a higher adhesion than titanium.

Figure 10 shows magnified fluorescence images of the endothelial cells over the patterns. We can appreciate how the cells were placed over one or more holes or in contact with them. In the case of the microstructures, we can observe that the endothelial cells, in their adhesion and spreading process, attached to these points. As we mentioned in the introduction, several works demonstrated that a surface modification on the micron scale affects the cell adhesion and can regulate cellular functions [40]. As we can appreciate in Figure 11, there was a local modification of the average roughness inside the microhole in comparison with the flat surface. Roughness is another physical factor that can control the cell adhesion. In conclusion, the fabrication of these structures alters the surface topography in the micron scale and also modifies the roughness inside the hole, leading microholes to act as anchor points for cells, increasing the number of cells adhered.

This work is a first approach to the biocompatibility of different structured material and to the cell responses to them. In the future, long-term biocompatibility tests could be performed in order to find the most suitable pattern for enhancing cell adhesion over different materials. The response of different kinds of cells over different substrates could also be evaluated.

## 5. Conclusions

In this work, a low cost method for fabricating periodic patterns over biocompatible materials (titanium and tantalum) has been presented. These surfaces have been microstructured in one single step by using the Talbot effect in combination with a microlens array. Cell adhesion of human umbilical vein endothelial cells over these microstructured materials has been studied. We found that the manufactured structures acted as anchor points for the cells, such that they preferentially adhered to the pattern and then spread toward other points or cells attached. In this first approach, we found that the 90 μm period pattern promoted cell adhesion on tantalum and the 45 μm period pattern did it on titanium.

## Figures and Tables

**Table 1:** Experimental parameters of the micro-patterns fabricated over titanium and tantalum.

	TITANIUM			TANTALUM		
	First Talbot Plane	3/2 Talbot Plane	5/3 Talbot Plane	First Talbot Plane	3/2 Talbot Plane	5/3 Talbot Plane
Microlens-substrate distance (mm)	16.21 ± 0.01	23.85 ± 0.01	26.42 ± 0.01	16.21 ± 0.01	23.85 ± 0.01	26.42 ± 0.01
Period (μm)	90.28 ± 0.10	45.14 ± 0.10	29.62 ± 0.10	89.84 ± 0.10	45.61 ± 0.10	29.92 ± 0.10
Spot diameter (μm)	16.15 ± 0.10	12.20 ± 0.10	5.52 ± 0.10	13.26 ± 0.10	10.77 ± 0.10	5.44 ± 0.10
Spot depth (μm)	3.1 ± 0.3	2.2 ± 0.3	0.8 ± 0.3	2.3 ± 0.3	1.7 ± 0.3	0.6 ± 0.3

All values indicate the mean of 10 measurements ± the accuracy of the measurement, since the standard deviation of the mean is negligible.
